# Autoimmune Cytopenias in Pediatric Hematopoietic Cell Transplant Patients

**DOI:** 10.3389/fped.2019.00171

**Published:** 2019-05-03

**Authors:** Jessica A. Neely, Christopher C. Dvorak, Matthew S. Pantell, Alexis Melton, James N. Huang, Kristin Ammon Shimano

**Affiliations:** Department of Pediatrics, University of California, San Francisco, San Francisco, CA, United States

**Keywords:** immune cytopenias, stem cell transplantation, post-transplant autoimmunity, autoimmune hemolytic anemia, immune thrombocytopenia, autoimmune neutropenia

## Abstract

**Background:** Autoimmune cytopenias (AICs) are potentially life-threatening complications following hematopoietic cell transplantation (HCT), yet little is understood about the mechanism by which they develop. We hypothesized that discordant B cell and T cell recovery is associated with AICs in transplant patients, and that this might differ based on transplant indication.

**Methods:** In this case control study of children who underwent HCT at our institution, we evaluated the clinical and transplant characteristics of subjects who developed AICs compared to a control group matched by transplant indication and donor type. In cases, we analyzed the state of immune reconstitution, including B cell recovery, T cell recovery, and chimerism, immediately prior to AIC onset. Subjects were stratified by primary indication for transplant as malignancy (*n* = 7), primary immune deficiency (PID, *n* = 9) or other non-malignant disease (*n* = 4). We then described the treatment and outcomes for 20 subjects who developed AICs.

**Results:** In our cohort, cases were older than controls, were more likely to receive a myeloablative conditioning regimen and had a significantly lower prevalence of chronic GVHD. There were distinct differences in the state of immune recovery based on transplant indication. None of the patients (0/7) transplanted for primary malignancy had T cell recovery at AIC onset compared to 71% (5/7) of patients with PID and 33% (1/3) of patients with non-malignant disease. The subset of patients with PID and non-malignant disease who achieved T cell reconstitution (6/6) prior to AIC onset, all demonstrated mixed or split chimerism. Subjects with AIHA or multi-lineage cytopenias had particularly refractory courses with poor treatment response to IVIG, steroids, and rituximab.

**Conclusions:** These results highlight the heterogeneity of AICs in this population and suggest that multiple mechanisms may contribute to the development of post-transplant AICs. Patients with full donor chimerism may have early B cell recovery without proper T cell regulation, while patients with mixed or split donor chimerism may have residual host B or plasma cells making antibodies against donor blood cells. A prospective, multi-center trial is needed to develop personalized treatment approaches that target the immune dysregulation present and improve outcomes in patients with post-transplant AICs.

## Introduction

Autoimmune cytopenias occurring after allogeneic HCT are well-described in the literature in both children and adults, but the pathophysiology remains poorly characterized (1–10). Clinically, these cytopenias tend to follow a more severe course and are more resistant to first-line treatments than in non-transplant pediatric patients with *de novo* autoimmune cytopenias, suggesting that the pathogenesis may be distinct (6, 8). Previous estimates of the incidence range from 2.5% in a large multicenter Italian study, to as high as 19.5% in a small cohort of patients with SCID, all of which manifested as autoimmune hemolytic anemia (AIHA) (3, 4). HCT from an unrelated donor, alemtuzumab serotherapy, CMV reactivation, and HCT for a primary non-malignant disease (particularly inborn errors of metabolism) have been described as risk factors for the development of AICs (3, 5, 6, 9, 11). Additionally, in a cohort of patients with Wiskott-Aldrich syndrome (WAS), mixed chimerism was associated with the development of autoimmunity in the post-transplant period with the most common manifestation being autoimmune cytopenias (12, 13). While T cell dysfunction has been posited as a possible mechanism for the development of AIHA in a cohort of 8 patients transplanted for SCID, the immune dysregulation present at the time of AIC onset is not well-understood (4). Characterizing the immune dysregulation that leads to autoimmune cytopenias is important both to optimize surveillance in the post-transplant period and to best target therapy. We sought to examine the state of immune reconstitution in a cohort of patients at our institution transplanted for a variety of diseases to determine the state of B and T cell recovery, the temporal relationship of B and T cell recovery, and chimerism status in patients who develop AICs.

## Methods

### Study Design and Subjects

This is a case control study of patients age 0–21 who underwent HCT at University of California, San Francisco (UCSF) Benioff Children's Hospital between 1/1/2000 and 7/1/2015 and were listed in the Bone Marrow Transplant Registry at UCSF. The study was approved by the UCSF Institutional Review Board. Cases were defined as any patient who received an allogeneic HCT and developed a post-transplant AIC, including autoimmune hemolytic anemia (AIHA), immune thrombocytopenia (ITP), autoimmune neutropenia (AIN), or multilineage cytopenias. Cases were identified by a variety of means to ensure capture including: ICD-9 codes for autoimmune cytopenias, pharmacy records for patients receiving intravenous immune globulin (IVIG) and rituximab on the hospital unit, and physician recall. Controls were identified from the same registry in a 2:1 ratio matched for primary disease category (malignancy, PID, or other non-malignant) and type of transplant (matched related, unrelated, or haploidentical donor). Subjects were sorted by these conditions and chosen randomly as controls. Exclusion criteria included death or transfer of care prior to collection of immune reconstitution data.

### Measures and Definitions

AICs were defined as follows: AIHA was defined by clinical evidence of hemolysis and a positive Coombs test; ITP was defined as a sudden drop in previously-normal platelet counts to <100 × 10^9^/L in the absence of another cause, consistent with the International Working Group definition (14); AIN was defined as a repeated ANC <500 cells/uL without other cause, and other supporting features including bone marrow examination demonstrating normocellular bone marrow and/or positive anti-neutrophil antibodies as agreed upon by 3/3 members of the research team. Several patients had multilineage cytopenias, and all of these were a combination of AIHA and ITP. Chronic graft-vs.-host disease (GVHD) was defined per the 2014 NIH consensus guidelines (15).

Immune reconstitution data was collected by retrospective chart review. Standard practice at our institution is to measure T and B cell subsets, IgM and IgA levels, and phytohemagglutinin (PHA) stimulation response starting at 3 months post-transplant and continuing every 1–3 months until in normal range per our institution's guidelines. T and B cell number, IgM and IgA levels, and PHA response were collected at the time-point most proximal to AIC onset, which could be before or after diagnosis of AIC, but prior to any immune suppressive therapy. Data were available for each subject, on average, within 28 days of AIC onset (range 0–86 days). B cell recovery was defined as CD19 count >50 cells/uL with IgM & IgA levels in the normal range for age. T cell recovery was defined as CD4 count >200 cells/uL and response to PHA stimulation >50% of control. These definitions and cutoffs for immune reconstitution are based on our institution's clinical Standard Operating Procedure for post-HSCT immune reconstitution (16). Lineage-specific chimerism, which is typically followed monthly for the first 6–12 months post-transplant at our institution, was also evaluated at the time point most proximal to AIC onset but prior to therapy. Lineage specific chimerism in controls was extracted at 5–6 months post-transplant, which was the median time of onset of AICs in cases. Full chimerism was defined as >95% donor in peripheral blood and mixed chimerism as 95% donor or less in peripheral blood. Split chimerism was defined as full chimerism in whole peripheral blood, but mixed chimerism, 95% donor or less, in one or more lineage.

### Statistical Analysis

Cumulative incidence of AICs was calculated for the entire patient population transplanted at our center over this time period as well as by transplant indication. Confidence intervals were calculated by the modified Wald method. A chi-squared test was used to determine if the proportion of subjects who developed AICs differed by transplant indication. Patient characteristics between cases and controls were compared by *t*-test for continuous variables and chi-squared test for categorical variables. Within cases, chi-squared test was used to compare the proportion of cases within each primary disease category (malignancy, PID, or non-malignant disease) who achieved T cell recovery, and this was repeated for B cell recovery. A proportion test was used to compare the proportion of cases and controls who achieved B cell recovery prior to T cell recovery (compared to coincident recovery or T cell recovery before B cell recovery). Wilcoxan signed-rank test was used to compare the paired data from cases of lineage-specific chimerism at AIC onset to last available chimerism. Wilcoxan rank-sum test was used to compare overall chimerism between cases at AIC onset and chimerism at 5–6 months (which is the median time to AIC onset in cases) in controls.

## Results

### Patient and Transplant Characteristics

The total incidence of AICs in all patients (*N* = 442) who underwent HSCT at UCSF during this time period was 4.5%. The incidence of AICs per primary indication was as follows: 6.0% in the PID group (95% CI 3.1–11.2%), 2.9% in the malignancy group (95% CI 1.3–6.1%), and 5.2% in the non-malignant disease group (95% CI 1.6–13.0%). There was no difference in the proportion of cases who developed AICs by transplant indication (*P* = 0.83). The characteristics of the cases and matched controls are shown in Table 1. Of cases, 45% (*N* = 9) had AIHA, 30% (*N* = 6) multilineage cytopenias, 15% (*N* = 3) ITP, and 10% (*N* = 2) AIN. Nine patients had PID, 7 had malignant disease, and 4 had an inborn error of metabolism or thalassemia. Median time to AIC onset was 5.2 months (range, 1.4–15.1 mos) post-HCT. Six out of 19 patients were on immune suppressant therapy at AIC onset (see Supplementary Table 1). Among variables tested in a univariate analysis, patients who developed AICs were significantly older than the control group (8.2 ± 7.3 yrs vs. 4.0 ± 4.7 yrs, *P* = 0.030). Only one patient (5%) in the study group developed chronic GVHD, compared to 11 patients (26%) in the control group (*P* = 0.048). There was a trend toward a higher proportion of cases receiving a myeloablative conditioning regimen and a higher proportion of controls receiving a non-myeloablative conditioning regimen (*P* = 0.08). There was no significant difference in gender, source of stem cells, ABO compatibility, anti-lymphocyte serotherapy, GVHD prophylaxis, prior donor lymphocyte infusion (DLI), total body irradiation (TBI), pre-transplant autoimmunity or prevalence of acute GVHD. Only 2 cases had pre-transplant autoimmunity, and only 1 had a pre-transplant cytopenia: patient 3 had acquired factor VIII, nephrotic syndrome, bullous pemphgoid, and inflammatory bowel disease, and patient 4 had ITP, enteritis, and type I diabetes mellitus (see Supplementary Table 1). Mortality rate for patients who developed AICs was double that of controls, 15% compared to 7%, however this did not meet statistical significance (*P* = 0.328). In the control cohort, the median time to chronic GVHD onset was 5.6 months (range, 1.6 mos-29.1 mos), similar to the median time to AIC onset (5.2 months), in the study cohort. Additional details regarding the primary indication for transplant in the control group are outlined in Supplementary Table 2.

**Table 1:** Patient characteristics matched by primary disease and HSCT.

**Characteristic**	**Study (*N* = 20)**	**Controls (*N* = 42)**	***P*-value**
Male	55% (11)	52% (22)	0.85
Age at transplant (yrs)	8.2 ± 7.3	4.0 ± 4.7	**0.03**
Primary disease			
Immunodeficiency	45% (9)	48% (20)	0.98
Malignant	35% (7)	33% (14)	
Non-malignant other	20% (4)	19% (8)	
Pre-transplant autoimmunity	10% (2)	5% (2)	0.50
Type of HSCT			
Related	15% (3)	14% (6)	0.99
Unrelated	60% (12)	62% (26)	
Haploidentical	25% (5)	24% (10)	
Source of cells		
Bone marrow	20% (4)	43% (18)	0.21
Peripheral blood	60% (12)	45% (19)	
Cord blood	20% (4)	12% (5)	
ABO donor/recipient			
Identical	75% (15)	57% (24)	0.32
Compatible (donor O+)	5% (1)	17% (7)	
Other combinations	20% (4)	26% (11)	
Conditioning regimen			
Myeloablative	75% (15)	62% (26)	0.08
Reduced Intensity	20% (4)	9% (4)	
Non-myeloablative	5% (1)	29% (12)	
Total body irradiation (TBI)	20% (4)	29% (12)	0.46
Anti-lymphocyte therapy (serotherapy)			
Alemtuzumab	45% (9)	31% (13)	0.28
ATG	50% (10)	50% (21)	
none	5% (1)	19% (8)	
GVHD prophylaxis			
TCD	30% (6)	24% (10)	0.92
CNI+MTX	40% (8)	47% (20)	
CNI+MMF	15% (3)	12% (5)	
Other (CSA ± prednisone)	15% (3)	17% (7)	
DLI prior to AIC onset	15% (3)	24% (10)	0.43
Acute GVHD Grade II-IV	35% (7)	26% (11)	0.48
Chronic GVHD	5% (1)	26% (11)	**0.048**

*ATG, anti-thymocyte globulin; GVHD, graft versus host disease; TCD, T-cell depletion; CNI, calcineurin inhibitor; MTX, methotrexate; MMF, mycophenolate mofetil; CSA, cyclosporin A; DLI, donor lymphocyte infusion. Bold values indicate significance of p-value < 0.05*.

### Immune Reconstitution

Among patients who developed AICs, there was a trend toward having B cell reconstitution but not T cell reconstitution at time of AIC onset, with 35% (6/17) having achieved T cell reconstitution and 69% (11/16) having achieved B cell reconstitution (*p* = 0.051), of those with available data. However, analysis of disease subgroups revealed notable differences: none of the patients with malignancy (0/7) had T cell recovery at AIC onset in contrast to 71% (5/7) of patients with PID, and 33% (1/3) of patients with other non-malignant disease (*P* = 0.020) (Table 2). B cells had fully recovered in 50% (3/6) of patients with malignancy, 75% (6/8) of patients with PID, and 100% (2/2) of patients with other non-malignant disease (*P* = 0.361) (Table 2). There was no difference between cases and controls in the proportion of subjects with B cell recovery prior to T cell recovery compared to coincident recovery or T cell recovery prior to B cell recovery. Of cases, 45% (5/11) had B cell recovery prior to T cell recovery compared to 38% (15/39) of controls (*P*=0.67), but this comparison was limited by the number of patients who achieved immune recovery prior to immune suppressive therapy.

**Table 2:** Immune reconstitution data of 20 subjects with post-transplant AIC stratified by primary disease.

**Primary disease**	**Pt ID**	**Mos post-transplant**	**B cell reconstitution**				**T cell reconstitution**			**Chimerism**		
			**CD 19+ B cells (×10^**6**^/L)**	**IgM (mg/dl)**	**IgA (mg/dl)**	**Recon-stituted**	**CD4+ T cells (×10^**6**^/L)**	**PHA**	**Recon-stituted**	**Overall**	**B cell (×10^**6**^/L)**	**T cell (×10^**6**^/L)**
Malignancy (*N* = 7)	11	4.4	284	19 (L)	98	No	520	L	No	Mixed	56	80
	17	6.8	75	42	96	Yes	88	L	No	Full	100	100
	8	4.8	68	23 (L)	57	No	110	L	No	Full	100	100
	12	3.8	205	48	87	Yes	42	–	No	Mixed	41	92
	15	6.3	81	–	–	No	70	L	No	Split	93	100
	16	5.3	97	128	–	–	139	L	No	Full	100	100
	7	13.6	70	40	77	Yes	434	L	No	Full	100	100
PID (*N* = 9)	13	4.2	114	103	55	Yes	4	–	No	Full	100	100
	9	5.2	40	20 (L)	620 (H)	No	281	L	No	Mixed	46	11
	3	8.9	578	58	180	Yes	456	N	Yes	Mixed	91	92
	4	13	626	61	57	Yes	908	N	Yes	Mixed	36	87
	5	8.1	83	31 (L)	–	No	415	N	Yes	Mixed	74	22
	2	15.1	1058	92	–	Yes	972	N	Yes	Mixed	11	31
	18	11.7	434	50	69	Yes	432	N	Yes	Mixed	63	14
	19	1.4	412	57	–	Yes	511	–	–	Full	100	100
	20	2.7	–	–	–	–	–	–	–	Split	99	19
Non-malignant (*N* = 4)	10	5.1	316	–	–	–	87	L	No	Full	100	99
	1	2.2	835	111	–	Yes	24	L	No	Split	100	83
	14	4.6	993	80	26	Yes	229	N	Yes	Split	99	88
	6	1.9	–	–	–	–	–	–	–	Split	91	38

*All values for IgM and IgA are in normal range for age unless otherwise indicated; – indicates data is not available for these values. Full chimerism: >95% donor in peripheral blood. Mixed chimerism: 95% donor or less in peripheral blood. Split chimerism: full chimerism in peripheral blood, but mixed chimerism, 95% or less, in one or more lineage. PHA, phytohemagglutinin*.

### Chimerism

Chimerism data were available at least 1 year post-transplant for all patients except one who died 6 months post-transplant of disease relapse. There was no significant difference between overall chimerism at AIC onset in cases compared to overall chimerism at 5–6 months in controls, though there was a trend toward lower donor chimerism in the T cell lineage in cases where median T cell chimerism was 83% compared to 97% in controls (*P* = 0.08). Lower T cell chimerism in cases was mostly driven by the PID group where the median T cell chimerism was 31%, compared to 100% in malignancy and 86% in the non-malignant group (Table 2). Evaluation of chimerism at AIC onset in cases compared to last available chimerism at data censorship showed no difference except in the T cell lineage where there was increasing T cell engraftment over time (*P* = 0.01). A similar pattern was observed in the control group where there was no difference in the B cell and myeloid chimerism at 5–6 months post-transplant and the last available chimerism, but there was increasing chimerism in the T cell lineage over time (*P* < 0.001).

Among cases, none of those who had achieved T cell reconstitution at time of AIC onset had achieved full chimerism. Conversely, no patients (one patient had incomplete data) who had full donor chimerism at time of AIC onset had achieved T cell reconstitution (Table 2). Mixed chimerism at time of AIC onset was observed in 40% (8/20) of patients overall, and split chimerism was observed in 25% (5/20). In cases, stratified by disease subgroup, mixed or split chimerism was present in 43% (3/7) of patients with malignancy, 78% (7/9) of patients with PID, and 75% (3/4) other non-malignant disease (*P* = 0.04).

### Outcomes

Eighty percent of patients resolved their AIC at a median time of 5 months from time of onset (range, 1–69 mos). The two subjects with isolated AIN resolved at 3 months and 12 months after onset, and the three subjects with isolated ITP resolved at approximately 3 months after onset. Those with AIHA or multilineage cytopenias had the widest range in time to resolution. Three patients died, 2 of malignancy relapse, and 1 directly of intractable hemolysis, representing a relative risk of death of 2.1 (95% CI 0.5–9.5) compared to the control group, which was not statistically significant.

As shown in Table 3, therapies for AICs included steroids, IVIG, rituximab, sirolimus, cyclophosphamide, danazol, bortezomib, DLIs, conditioned stem cell boost, and second transplant in the most extreme case. Patient 2, who had AIN, also had falling myeloid chimerism, so first line therapy was deferred while giving DLI in hopes that improving chimerism might also ameliorate his cytopenia and avoid need for concomitant steroids with DLI, a strategy reflected in the literature but not standard for AICs (17). Fifteen of twenty (75%) patients required addition of a 2nd or 3rd line agent for inadequate response to initial treatment or relapse of hemolysis. Patients with AIN or ITP tended to respond to a combination of IVIG and steroids +/– rituximab. However, the majority of patients with AIHA or multilineage cytopenias did not have complete response after treatment with steroids, IVIG and/or rituximab. Of the five patients treated with steroid +/– IVIG as initial therapy, only one patient had sustained complete AIC remission.

**Table 3:** Treatment and outcomes.

**Pt ID**	**Type of AIC**	**Primary disease**	**Therapy given**			**Time to resolution or death (mos)**	**Mortality**
			**1st line**	**2nd line**	**3rd line & additional**		
11	AIHA	Hodgkin lymphoma	Rituximab	Sirolimus		8	Living
17	AIHA	CML	Rituximab + steroids			8	Living
8	AIHA	T-cell ALL	Steroids	Rituximab	Sirolimus	5	Living
12	AIHA, ITP	HR B-cell ALL	Steroids + rituximab + bortezomib	Conditioned stem cell boost (CYC and ATG)	Sirolimus + ibrutinib switched to daratumumab	>17	Living
15	AIHA, ITP	AML (M6)	Steroids			6	Deceased; AML relapse & pneumonia
16	AIHA, ITP	MDS	Steroids + rituximab	Bortezomib		23	Deceased; disease relapse
7	AIHA, ITP	T-cell ALL	Rituximab	Steroids + rituximab	Steroids + rituximab + IVIG, then sirolimus added	24	Living
13	AIHA	SCID	Steroids, IVIG, ritux	Danazol	PEX followed by 4th transplant	5	Deceased; ARDS & intractable hemolysis
9	AIHA, ITP	SCID	IVIG (for isolated ITP)	Sirolimus added for AIHA	Stem cell boost	3	Living
3	AIHA	ZAP-70 deficiency	Steroids + rituximab	Steroids	Steroids + sirolimus	69	Living
4	AIHA	IPEX	Steroids + rituximab	High-dose CYC	Rituximab, steroids and sirolimus	32	Living
5	AIHA	SCID	Steroids + rituximab	IVIG, steroids, rituximab, azathioprine	High-dose CYC, IVIG, rituximab	21	Living
2	AIN	CGD	DLI x3	Steroids	IVIG + rituximab	12	Living
18	AIHA, ITP	SCID	Steroids	Rituximab		>26 & <38^*^	Living
19	AIHA, ITP	WAS	Steroids + IVIG	Rituximab		2	Living
20	ITP	CD40L deficiency	Steroids + IVIG			3	Living
10	AIHA	Aplastic Anemia (SDS)	Steroids			1	Living
1	ITP	Beta thalassemia major	IVIG	Rituximab		2	Living
14	AIN	Hurler Syndrome	GCSF			3	Living
6	ITP	Hurler Syndrome	Steroids + IVIG	Rituximab		3	Living

* *Patient lost to follow up for 1 year and cytopenia resolved during this time period during which cytopenia resolved. CML, chronic myelogenous leukemia; ALL, acute lymphoblastic leukemia; HR, high risk; CYC, cyclophosphamide; ATG, anti-thymocyte globulin; AML, acute myelogenous leukemia; MDS, myelodysplastic syndrome; IVIG, intravenous immune globulin; SCID, severe combined immunodeficiency; PEX, plasma exchange; ARDS, acute respiratory distress syndrome; IPEX, immunodysregulation polyendocrinopathy enteropathy X-linked; CGD, chronic granulomatous disease; DLI, donor lymphocyte infusion; WAS, Wiskott-Aldrich syndrome; CD40L, CD40 ligand; SDS, Shwachman-Diamond syndrome; GCSF, granulocyte colony-stimulating factor*.

The nine patients with AIHA or multilineage cytopenias treated with rituximab as part of initial treatment had a more refractory course requiring addition of other therapies. Patients 3, 4, 5, 12, and 7 represent the most refractory cases with duration of disease ranging from 21 to 69 months. Patients 3, 4, and 7 ultimately had resolution with addition of sirolimus and patient 5 had response with high-dose cyclophosphamide. Patient 12 had an especially refractory course, with ongoing hemolysis after steroids, rituximab, bortezomib, stem cell boost conditioned with cyclophosphamide and ATG, sirolimus, ibrutinib, and daratumumab with ongoing hemolysis up until the time of data censorship for this analysis [this patient subsequently died due to infectious complications prior to publication of this manuscript (18)].

## Discussion

This study describes the immune reconstitution patterns and outcomes of 20 pediatric patients who developed AICs following HCT. Compared to non-affected transplant patients, those who developed AICs were more likely to have T cell dysfunction at the time of AIC onset. These results are consistent with previous observations at our institution by Horn et al. who demonstrated T cell dysfunction in a cohort of eight SCID patients manifested as low CD4+ or CD8+ count or poor response to PHA (4). Likewise, a recent study demonstrated skewed Th2 response in patients with post-transplant AICs as well as lower CD3+CD8+ T cells compared to a control group (11). While the pathogenesis of post-transplant AICs is likely multifactorial, these findings suggest that T cell dysfunction leading to autoantibody production by partially-functional but dysregulated B cells may be one contributing mechanism. Interestingly, this phenomenon was most pronounced in patients transplanted for malignancy. We found significant differences in immune profiles based on primary transplant indication. Patients with malignancy uniformly had T cell dysfunction at AIC onset, compared to only a subset of those with PID or non-malignant disease. While these data are purely descriptive, we speculate that in patients without malignancy, other mechanisms may also contribute to the pathogenesis of AICs. Possibilities include the persistence of autoreactive host lymphocytes or aberrant development of double-negative CD3+ cells, and these represent an area for future research.

In a large cohort of patients with Wiskott-Aldrich syndrome, patients who developed autoimmune manifestations post-transplant demonstrated lower donor chimerism in all three cell lineages than patients who did not develop autoimmune manifestations (12, 13). In our study, the majority of patients with PID or other non-malignant diseases had mixed or split chimerism at the time of AIC onset, while patients with malignancies were more likely to be full donor chimeric. This finding likely reflects differing transplant strategies but suggest that chimerism may also be contribute to post-transplant autoimmunity in certain patient types. Patients who do not have full donor T cell chimerism may have residual host lymphocytes causing autoimmunity, whereas patients who have full donor chimerism but have poor T cell reconstitution may be unable to properly regulate the antibody-producing autoreactive B-cells. It should be noted that chimerism was evaluated relatively early in the post-transplant period given the median time to AIC onset was 5.6 months. Comparing engraftment at AIC diagnosis with last available engraftment data at time of data censorship, we found no difference in lineage-specific engraftment, except in the T cell lineage where engraftment increased over time. This finding may indicate a role for poor T cell chimerism in AICs or reflect the natural history of increasing engraftment over time as a similar pattern was observed in the control group.

Because prior studies have identified primary non-malignant disease and transplant from an unrelated donor as risk factors for AIC development in the post-transplant period, we chose to match our control group for primary disease and donor type (3, 6, 9). Due to this design, we are unable to comment on transplant indication or donor type as a risk factor for AICs, though we did not see a difference in the incidence of AICs by primary indication when examining our entire cohort. In matching for these variables, we found that patients who developed AICs were older at time of HSCT and had a lower incidence of chronic GVHD than controls. The difference in age was in contrast to a Minnesota study which found that patients in their cohort aged <10 years were more likely to develop autoimmune cytopenias than patients aged 10–17 years (6). Likewise, an Italian study found younger age to be a significant risk factor in a univariate analysis with median age of 3.1 years in patients who developed AICs, but no effect of age in a multivariate analysis (3). We speculate that older patients may be at increased risk for development of AICs due to increasing thymic involution and dysfunction with age, but we believe the exact role of age on the development of post-transplant AICs requires further study, ideally in a multi-center fashion to control for local biases in patient characteristics.

Additionally, neither the Minnesota study or the Italian study found a difference in the development of chronic GVHD between patients with AIC and unaffected patients. While the incidence of chronic GVHD was very low in our population of patients with AICs, 26% of our control population developed chronic GVHD, which is on par with other rates of cGVHD reported in the malignant (19) and non-malignant populations (20). The median time to onset AIC in our study population was similar to the median time to development of chronic GVHD in our control group, occurring between 5 and 6 months. Additionally, the median time to AIC resolution of 5 months in our cases is similar to the time to cGHVD resolution seen in a cohort of 121 cGVHD patients (21). As others have suggested, we propose that in some patients, AICs may simply be a hematologic manifestation of chronic GVHD (7). B cell dysregulation has been implicated in the pathophysiology of cGVHD, providing a possible overlapping mechanism between AICs and cGVHD (22, 23). Current NIH consensus criteria regarding diagnosis and staging of chronic GVHD for clinical trials recognize hematopoietic and immunological abnormalities, including AIHA and ITP, as common associations with chronic GVHD but do not consider the presence of these abnormalities alone to be diagnostic of chronic GVHD (15). Our results suggest it may be reasonable to consider autoimmune cytopenias as a diagnostic hematological manifestation of chronic GVHD. Alternatively, it is possible that treatment of AICs with steroids, rituximab, and other immunosuppressants might have abrogated the later development of chronic GVHD.

Consistent with prior literature, we found AICs to be refractory to first-line treatment, with only 25% demonstrating response to initial therapy. Furthermore, our cohort demonstrated an overall poor response to rituximab. B-cell directed therapies such as rituximab treat one aspect of the pathogenesis of the AIC by attacking the presumed clonal population of B cells that is mediating the autoimmunity. If residual host plasma cells are responsible for the dysregulated antibody production, however, targeting CD20 is insufficient, and therapies such as bortezomib (24) or daratumumab (18) may be necessary. When these abnormal populations of B cells are permitted to flourish in the setting of T-cell dysregulation or insufficiency, however, the addition of sirolimus may help to allow for the preferential survival and expansion of regulatory T cells, restoring control over the autoreactive cells and permitting development of tolerance. It is possible that this therapy may be efficacious both in settings of mixed T cell chimerism with residual host lymphocytes and in incomplete T-cell reconstitution with a paucity of regulatory T cells. There is growing evidence to support the use of sirolimus in AICs outside of the transplant setting (25), and our data would support a mechanistic rationale for its use in post-transplant cytopenias. A formal trial of early sirolimus use in patients with post-transplant AIC is needed.

There are several limitations to this study, including the retrospective nature, small study size, difficulty in identifying patients with isolated ITP or AIN due to lack of a single diagnostic laboratory test, and incomplete immunologic data at time of AIC diagnosis. Nonetheless, these results highlight T cell dysregulation as a possible mediator of AICs, at least in patients with malignancy and in some patients with PID. Given the heterogeneity of AICs and the treatment implications of elucidating the pathophysiology, we recommend further characterization of the immune dysregulation present in each individual patient at time of AIC diagnosis. A comprehensive immunologic evaluation including lymphocyte subsets with CD3+, CD4+, CD8+, CD19+ counts, naïve and memory T cells, regulatory T cell subsets, B-cell phenotyping, PHA, and total IgA and IgM may help clarify the underlying immune derangement and guide a mechanism-based decision to use T- or B-cell directed therapy (or both). Lastly, we feel that a collaborative prospective study of autoimmune cytopenias is necessary to understand the true incidence and better characterize the risk factors and immune dysregulation that lead to this post-transplant complication. This knowledge could potentially equip physicians to choose a more effective therapy at AIC onset to decrease the overall morbidity and mortality associated with AIC's in the post-transplant pediatric population.

## Ethics Statement

This study was approved by the University of California, San Francisco Institutional Review Board and was declared to be exempt. No written consent was required given this study required only medical record review and all identifiable data is anonymized.

## Author Contributions

JN, KS, and CD contributed to the conception and design of the study. MP and JN contributed to the statistical analysis of the study. JN, KS, CD, JH, and AM contributed to the interpretation of data. JN wrote the initial manuscript draft. KS, CD, JH, AM, and MP critically revised and edited the manuscript. All authors contributed to manuscript revision, read and approved the submitted version.

### Conflict of Interest Statement

JH receives research funding through Novartis, however, this funding was not used to support this research. The remaining authors declare that the research was conducted in the absence of any commercial or financial relationships that could be construed as a potential conflict of interest.
