# Supplementary material for: Autoimmune Cytopenias in Pediatric Hematopoietic Cell Transplant Patients
Source: Front Pediatr. 2019 May 3;7:171. doi: 10.3389/fped.2019.00171 (PMC6509944; doi:10.3389/fped.2019.00171)
Supplement: Supplementary file 1 [file Table_1.DOCX]

| **Supplementary Table 1: Additional patient characteristics of cases** | | | | |
| --- | --- | --- | --- | --- |
| **Patient ID** | **Primary Disease** | **Pre-transplant Autoimmunity** | **Immune Suppression at AIC onset** | **Isohematologic characterization** |
| 11 | Hodgkin Lymphoma |  | none | IgG+ (1+) |
| 17 | Chronic myeloid leukemia |  | cyclosporine | C3+(<1+) |
| 8 | T cell ALL |  | none | IgG+(<1+)/C3+(<1+) |
| 12 | B cell ALL |  | none | IgG(2+)/C3+(3+) |
| 15 | Acute myeloid leukemia (M6) |  | none | C3+(<1+) |
| 16 | Myelodysplastic syndrome |  | not available | NA |
| 7 | T cell ALL |  | prednisone | IgG+(1+) anti-e |
| 13 | SCID (X-linked) |  | none | IgG(2+)/C3+(1+) |
| 9 | SCID (Artemis) |  | none | IgG+(2+)/C3+(<1+) |
| 3 | ZAP-70 Immunodeficiency | nephrotic syndrome, acquired Factor VIII deficiency, bullous pemphigoid, IBD | none | IgG(2+)/C3+(1+) |
| 4 | IPEX syndrome | ITP, enteritis, type 1 diabetes mellitus | prednisone | IgG(2+)/C3+(1+) |
| 5 | SCID (DNA-PKcs) |  | cyclosporine, mycophenolate | IgG+(2+)/C3+(2+) |
| 2 | Chronic granulomatous disease |  | cyclosporine | NA |
| 18 | SCID (unknown mutation) |  | none | IgG+ (1+)/C3+ (1+) |
| 19 | Wiskott Aldrich Syndrome |  | cyclosporine, prednisone | IgG+ (2+) |
| 20 | CD40 ligand deficiency |  | none | C3+(<1+) |
| 10 | Aplastic anemia |  | none | IgG+(1+)/C3+(<1+) |
| 1 | Beta Thalassemia major |  | none | NA |
| 14 | Hurler Syndrome |  | none | NA |
| 6 | Hurler Syndrome |  | none | NA |
| *Abbreviations: ALL (acute lymphoblastic leukemia), SCID (severe combined immunodeficiency), IPEX (immune dysregulation, polyendocrinopathy, enteropathy, x-linked syndrome), IBD (inflammatory bowel disease* | | | | |
