# Supplementary material for: Autoimmune Cytopenias in Pediatric Hematopoietic Cell Transplant Patients
Source: Front Pediatr. 2019 May 3;7:171. doi: 10.3389/fped.2019.00171 (PMC6509944; doi:10.3389/fped.2019.00171)
Supplement: Supplementary file 2 [file Table_2.DOCX]

| **Supplementary Table 2: Characteristics of control group** | | | |
| --- | --- | --- | --- |
| Primary Disease | Donor type | Cell origin | Conditioning Regimen |
| Myeloid sarcoma/AML | haplo | PB | MA |
| Hodgkin's lymphoma | related | PB | MA |
| Acute lymphoblastic leukemia | related | PB | NMA |
| Acute lymphoblastic leukemia | haplo | PB | MA |
| Chronic myelogenous leukemia | unrelated | PB | MA |
| Acute lymphoblastic leukemia | unrelated | PB | MA |
| Acute myeloid leukemia | unrelated | PB | MA |
| Acute myeloid leukemia | haplo | PB | RIC |
| Acute lymphoblastic leukemia | related | BM | MA |
| Juvenile myelomonocytic leukemia | unrelated | CB | MA |
| Acute myeloid leukemia | unrelated | PB | MA |
| T cell lymphoma | related | BM | MA |
| Myelodysplastic syndrome | unrelated | PB | MA |
| Myelodysplastic syndrome | haplo | PB | MA |
| SCID, x-linked | haplo | PB | NMA |
| SCID, IL-7RA mutation | haplo | PB | NMA |
| Hemophagocytic lymphohistiocytosis | haplo | PB | MA |
| Acquired aplastic anemia | related | BM | NMA |
| Wiskott-Aldrich syndrome | unrelated | BM | MA |
| Wiskott-Aldrich syndrome | unrelated | PB | RIC |
| X-linked lymphoproliferative disease | unrelated | BM | MA |
| SCID, ADA def | unrelated | BM | NMA |
| Hemophagocytic lymphohistiocytosis | unrelated | BM | MA |
| SCID, IL7R | unrelated | BM | NMA |
| SCID, x-linked | haplo | PB | NMA |
| SCID, IL7R | haplo | PB | NMA |
| SCID, IL7R | haplo | PB | NMA |
| Cartilage-hair hypoplasia | related | BM | MA |
| SCID, IL-2R | unrelated | BM | RIC |
| Wiskott-Aldrich syndrome | unrelated | BM | MA |
| SCID, x-linked | unrelated | BM | NMA |
| Chronic granulomatous disease | unrelated | PB | MA |
| Hemophagocytic lymphohistiocytosis | unrelated | BM | MA |
| SCID, T-B+ | unrelated | BM | NMA |
| Hurler's syndrome | unrelated | BM | NMA |
| Hunter syndrome | unrelated | CB | RIC |
| Hurler's syndrome | unrelated | BM | MA |
| Alpha thalassemia major | related | CB | MA |
| Alpha-mannosidosis | unrelated | BM | MA |
| Fanconi's anemia | unrelated | CB | MA |
| Hurler's syndrome | unrelated | CB | MA |
| Hurler's syndrome | unrelated | BM | MA |
| *Abbreviations: SCID (severe combined immunodeficiency), AML. (acute myelogenous leukemia), NMA (non-myeloablative), MA (myeloablative), RIC (reduced intensity conditioning), PB (peripheral blood), BM (bone marrow), CB (cord blood)* | | | |
